# Characterization of the Diversity and Temporal Stability of Bacterial Communities in Human Milk

**DOI:** 10.1371/journal.pone.0021313

**Published:** 2011-06-17

**Authors:** Katherine M. Hunt, James A. Foster, Larry J. Forney, Ursel M. E. Schütte, Daniel L. Beck, Zaid Abdo, Lawrence K. Fox, Janet E. Williams, Michelle K. McGuire, Mark A. McGuire

**Affiliations:** 1 Department of Animal and Veterinary Science, University of Idaho, Moscow, Idaho, United States of America; 2 Department of Biological Sciences, University of Idaho, Moscow, Idaho, United States of America; 3 Department of Statistics, Department of Mathematics, University of Idaho, Moscow, Idaho, United States of America; 4 Department of Veterinary Clinical Science, Washington State University, Pullman, Washington, United States of America; 5 School of Biological Sciences, Washington State University, Pullman, Washington, United States of America; 6 Initiative for Bioinformatics and Evolutionary Studies (IBEST), University of Idaho, Moscow, Idaho, United States of America; Technion-Israel Institute of Technology, Israel

## Abstract

Recent investigations have demonstrated that human milk contains a variety of bacterial genera; however, as of yet very little work has been done to characterize the full diversity of these milk bacterial communities and their relative stability over time. To more thoroughly investigate the human milk microbiome, we utilized microbial identification techniques based on pyrosequencing of the 16S ribosomal RNA gene. Specifically, we characterized the bacterial communities present in milk samples collected from 16 women at three time-points over four weeks. Results indicated that milk bacterial communities were generally complex; several genera represented greater than 5% of the relative community abundance, and the community was often, yet not always, stable over time within an individual. These results support the conclusion that human milk, which is recommended as the optimal nutrition source for almost all healthy infants, contains a collection of bacteria more diverse than previously reported. This finding begs the question as to what role this community plays in colonization of the infant gastrointestinal tract and maintaining mammary health.

## Introduction

Due to the considerable health benefits it confers, human milk is universally considered the optimal source of nutrition for almost all healthy infants. For instance, breastfeeding provides infants with critical protection from diarrheal [1] and respiratory diseases [2], especially in developing countries, and is associated with reduced long-term risk of obesity [3], [4]. Past research [5], [6] has extensively investigated the presence and health implications of the traditional nutrients in milk, such as fatty acids, vitamins, and minerals; however, recent work has shown that human milk also contains communities of bacteria [7], [8], [9], [10], [11], [12] that may have health implications.

Culture-dependant methods have long confirmed the presence of bacteria in aseptically collected milk including *Staphylococcus* and *Streptococcus* species [7], whereas culture-independent studies utilizing microbial characterization techniques based on the amplification of bacterial 16S rRNA have shown that human milk contains several additional genera of bacteria including *Lactobacillus* and *Bifidobacterium* [8], [9], [10]. While these studies provide clear evidence that aseptically collected milk contains bacteria, very little work has examined the possibility that a core milk microbiome exists among lactating women, or investigated the stability of these communities within an individual over time. These types of analyses are critical because they make it possible to determine the roles these communities may play in maintaining mammary gland health, bacterial colonization of the infant's gastrointestinal tract, and other indices of short- and long-term maternal and infant health. Consequently, the present study was designed to probe more deeply into the stability and diversity of human milk bacterial communities over time. We hypothesized that human milk contains a greater diversity of bacterial phylotypes than previously noted, and that these communities would be stable over time within each individual lactating woman.

## Results

With the exception of milk collected from one participant who donated only 2 samples, bacterial genomic DNA was extracted from milk samples collected at 3 time points over a 4-wk interval from 16 lactating women self-described as healthy and free from lactational mastitis. Samples were collected using a method designed to reduce skin contamination. The V1-V2 region of the bacterial 16S rRNA gene was amplified from the DNA using universal primers, and barcoded pyrosequencing of the amplicons produced approximately 300,000 reads. Conservative quality control measures were employed to remove sequences with potential error such as those that had ambiguous bases, did not match the forward primer sequences, failed to align correctly to an established 16S rRNA sequence database, or were flagged as potential chimeras. These quality control measures reduced the data set to ∼160,000 high quality sequences with a mean of 3400 sequences per sample. To examine the bacterial genera present, sequences were then assigned to the most likely bacterial genera using the Ribosomal Database Project (RDP) Bayesian classifier.

**Figure 1:**
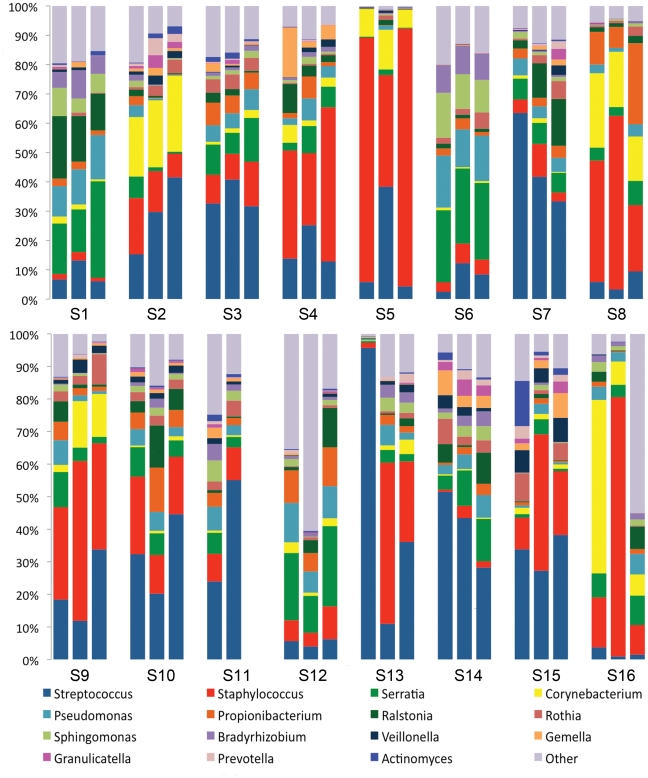
The community composition of the 15 most abundant bacterial genera in each of 3 milk samples from 16 subjects was diverse. The communities observed were found to be reasonably complex, and while consistent in composition over time for some subjects, a great deal of variation was observed over time in the samples of others.

**Figure 2:**
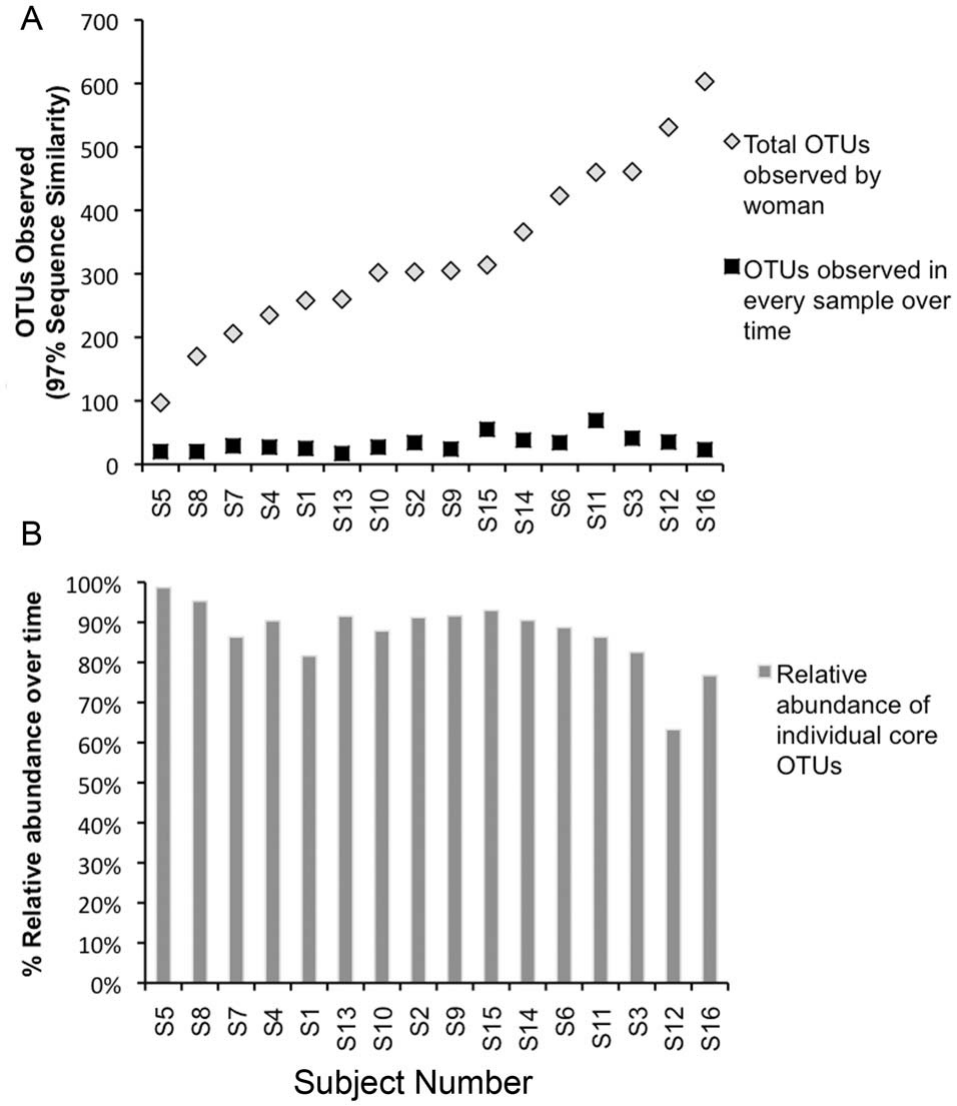
A small proportion of the total richness was persistent within subject and represented the majority of the community present. **A.** The number of OTUs (as defined by 97% sequence similarity) observed across all samples for each woman ranged from 100 to 600; however, only a small proportion of those OTUs were present in every sample from an individual subject-representing the individual core milk microbiome. **B.** The individual core milk microbiome for each woman was composed of the OTUs present in each of her samples. This relatively small number of OTUs represented the majority of the relative abundance of the community observed over time.

The most abundant genera in milk (Figure 1; Table S1) were *Streptococcus*, *Staphylococcus*, *Serratia* and *Corynebacteria*; however, eight other genera represented ≥1% of the communities observed across samples. Additionally, assignment of sequences into operational taxonomic units (OTUs) using a 3% similarity cutoff identified 100–600 OTUs present in the samples from each subject (Figure 2).

**Figure 3:**
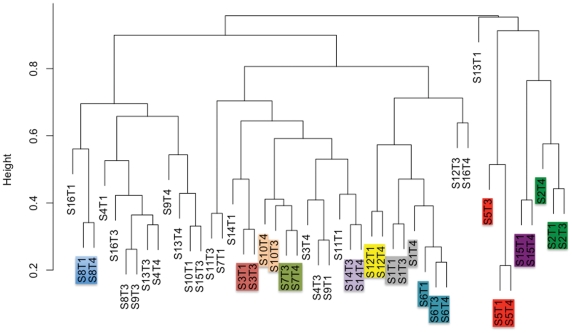
The community structure of bacterial OTUs in a milk sample often aligned by subject. The complete linkage clustering of the samples based on the Bray-Curtis similarity metric demonstrated that, with several exceptions, samples from the same woman were often most similar to other samples from that same subject. Colored boxes represent samples from the same subject that clustered together.

The variation among samples was studied to evaluate the stability (or lack thereof) of the communities within women. Examination of the communities on a sample-by-sample basis within subject suggested that the stability and membership of bacterial communities present were different among women (Figure 1; Table S1). For example, in each of the samples from “Subject 5,” *Staphylococcus* was either the first or second most abundant genera in the milk representing 22–59% of her bacterial community. In contrast, in all 3 samples collected from “Subject 1,” *Staphylococcus* was only a minor contributor to the community, consistently composing <5% of the bacteria therein. In samples from some individuals the milk bacterial communities were consistent and relatively unchanging over time (e.g., subjects 1 and 3); for others there was little stability as the relative abundance of the bacterial genera present shifted over time (e.g., subjects 13 and 16). Cluster analysis comparing the community structure of each sample (Figure 3) demonstrated a range of similarity in samples across subjects. All samples from four of the subjects clustered with each other, two samples from five subjects clustered directly together, whereas none of the samples from six of the subjects clustered together. Of the total OTUs observed in the samples from each woman only 4–20% were present in every milk sample collected from that woman over time (Figure 2). However, these few persistent OTUs dominated the community, representing between 60–99% of the bacterial abundance in samples from a particular woman.

Among subject variation was apparent in the relative abundances of bacterial genera (Figure 1; Table S1) and the six-fold difference in number of observed OTUs (Figure 2). In samples from several subjects *Streptococcus* was considerably more abundant than any other genera, whereas in samples from several other women *Staphylococcus* was the most abundant genera, and in samples from the remaining women no genera were consistently most prevalent. However, a set of 9 OTUs (Table 1) was found to be present in every sample from every subject. This small proportion of the overall membership of the milk microbiome represented a reasonably large (50%) proportion of the relative abundance in the total communities of the 16 subjects.

**Table 1:** Genus assignments of the 9 OTUs identified in every sample (*n* = 47) and their relative abundance (%).

Core OTU Genera	Relative abundance of OTU in total community (%)
*Staphylococcus*	15.8
*Streptococcus*	8.2
*Serratia*	7.6
*Pseudomonas*	4.5
*Corynebacterium*	3.8
*Ralstonia*	3.7
*Propionibacterium*	3.6
*Sphingomonas*	2.4
*Bradyrhizobiaceae*	1.9
Sum of all “core” OTUs	51.5

## Discussion

Previous culture-based and culture-independent studies have established that human milk contains several species of bacteria that are potentially important in maternal or infant health [7], [11], [12]. We extended these studies by performing an in-depth analysis of the bacterial communities in milk with high throughput sequencing techniques. The methods used in this study allowed us to carry out a more thorough examination of the biodiversity present in our samples by employing primers that are designed to be “universal” in nature, or capable of detecting most bacteria present in clinical samples [13]. Additionally, this type of technology facilitated the analysis of thousands of sequences per sample, which increased the capacity to observe less abundant bacterial phylotypes. The results of this study, therefore, provided a more comprehensive view of the ecology of milk bacterial communities.

Based on this difference in methodological approach it is reasonable that our results identified a much greater diversity of bacteria in milk than what has previously been reported in culture independent studies that relied on less broad range (quantitative PCR) [10] or precise (PCR-DGGE) [8] methods. Confirming previously published reports of milk microbiota [7], [10], we found several genera of bacteria in every sample, and the major phylotypes observed were *Streptococcus* and *Staphylococcus*. Additionally, as also reported in another analysis of milk bacteria that employed universal primers [8], we detected *Serratia* and *Propionibacterium* in the milk samples we analyzed. Conversely, whereas previous work has identified *Lactobacillus* and *Bifidobacteria* as common but minor members (2–3% relative abundance) of milk microbiota [9], [10], very few sequences from these phylotypes were observed in our samples (Table S1). This difference may be attributable to genetic, cultural, environmental, or dietary differences among subjects, especially considering that the previous studies were performed in Europe and the current study in the US. Additionally, differences in the primers used may be responsible for these conflicting findings.

An analysis of the microbial community membership across all the samples from the 16 subjects suggests that a “core” milk microbiome was present. Of the hundreds of OTUs detected in the milk of every woman, only 9 were present in every sample from every woman. Surprisingly, these 9 “core” OTUs represented about half of the microbial community observed, although the relative abundance of these 9 core OTUs varied greatly between subjects. Of course, that means that the remaining half of the community was not conserved across women. This is in stark contrast to the gut microbiome, where no highly abundant set of OTUs is shared among individuals [14], or the vaginal microbiome which comprises several different core groups [15]. In addition, studies of the various sites of the human microbiome indicate that the bacterial communities associated with a particular individual over time are often highly personalized [16]. This was also true of the human milk microbiome observed in this study; persistent OTUs present in every sample from an individual represented a large proportion of the abundance of the bacterial community observed.

The origin of the bacterial communities inhabiting milk is unknown. However, utilizing infrared photography, Ramsay et al. [17] demonstrated that a high degree of retrograde flow back into the mammary ducts occurs during suckling. This back flow may provide an ideal route for the exchange of bacteria from the infant's mouth into the mammary gland. Indeed, ecological niches in the human microbiome are not thought to be isolated environments, but rather a network of inter-related communities experiencing constant exchange [16]. It is likely that milk bacterial communities are no exception, and that they are constantly influenced by exposure to the other microbial populations associated with the mother and her infant. Little is known about the salivary microbiome of infants, but investigations of the adult salivary microbiome have demonstrated that *Streptococcus* species are the dominant phylotype therein [18], [19]. As previously noted, *Streptococcus* species were the most abundant phylotype in the milk samples we analyzed, supporting the hypothesis that the infant salivary bacteria play a role in establishing milk bacterial communities, or vice versa.

Several of the phylotypes typically present on adult skin such as *Staphylococcus*, *Corynebacteria*, and *Propionibacteria* [20], [21] were found in the milk of every woman. This presents the possibility that skin bacterial communities may be another source for the origin of milk bacteria and that interaction with the maternal skin microbiota may also help shape the distribution of milk microbiota. However, because we recognized the possibility that skin microbiota could contaminate our samples, the breast was cleansed with an iodine-based solution prior to milk sample collection. Additionally, a comparison of the bacterial communities we detected in milk to those of sebaceous skin of the type found on the breast indicates that although the two communities share many of the same phylotypes, major differences exist. Most notably, the genus found in greatest abundance in these milk samples (*Streptococcus*) is only a minor component of sebaceous skin microbiota, representing <10% of the genera found there [20], [21]. Likewise, *Propionibacteria* has been reported to be the most abundant genus on sebaceous skin, but this phylotype was not among the 5 most abundant genera in our milk samples. If the bacterial communities detected in our milk samples were merely due to skin contamination, then these communities would not only be expected to share several phylotypes, but the relative abundance of these phylotypes would also be similar. This difference between the two communities suggests that bacterial communities in milk are not simply a result of skin contamination.

The characteristics of commensal communities of bacteria in human milk with the level of complexity and individuality observed in our study may have important implications for the mammary health of lactating women. During the course of lactation, up to 30% of women suffer from breast infections or inflammation (mastitis), often leading to fever, redness, swelling, and breast pain [22]. Interestingly, whereas many women who experience mastitis do so repeatedly, others report no such problems throughout the course of several lactations [23]. It is possible that the composition of mammary communities, which the present data show are often unique to an individual, are important factors that influence whether a woman will suffer reoccurring episodes or avoid mastitis altogether. It is possible that mechanisms such as competitive exclusion for nutrients and other resources, or production of bacteriocins by particular members of the commensal communities in milk repress potential pathogens and the subsequent signs and symptoms of mastitis [7].

The pathogenic agent most commonly associated with lactational mastitis is *Staphylococcus aureus* [24], although often cases are considered “noninfectious” due to the lack of pathogen confirmation via the widely utilized, culture-based analyses [25]. We believe these conclusions may have been influenced by methodological bias. In support of this, we analyzed the milk samples collected in the present study using culture-dependent techniques to highlight the difference between what culture-independent methods were capable of, and what is usually determined with the industry standard for analysis of pathogens associated with mastitis. The method that was employed is currently recommended by the National Mastitis Council Laboratory and Field Handbook on Bovine Mastitis [26], which aims to determine the presence or absence of a pathogen. Briefly, samples of milk were cultured aerobically on blood agar plates and checked for growth at 24 and 48 h. Isolates were then identified as coagulase-positive or -negative *Staphylococci*, *Streptococcus*, *Cornybacterium* or coliforms. The interesting outcome of this analysis was that even with this simple method of screening for bacterial growth the shortcomings of culture based methods for analysis of milk bacteria became apparent when roughly 20% of samples were void of bacterial growth. The culture conditions used should support the growth of a variety of organisms, yet growth from these samples was limited. Therefore, this example of a basic culture analysis supports the conclusion that culture-independent methods provide valuable additional insight into the bacteriology of milk.

For example, although the women who participated in this study defined themselves as free from lactational mastitis, an intriguing microbial pattern was observed in the first sample from subject 13 (Figure 1). This sample was dominated by an apparent bloom of *Streptococcus*, which represented 95% of the total bacterial relative abundance. In contrast, the 2 other samples collected from this same woman displayed a more even phylotype distribution. Interestingly, at the time the *Streptococcus*-dominated sample was obtained, the somatic cell count of the sample, which is a measure of mammary gland inflammation commonly used in the dairy industry to detect mastitis [27], was five-fold greater when compared to the other two samples collected from the same subject. This drastic increase in a marker of inflammation suggests that acute lactational mastitis was likely occurring at the first time point, and provides evidence that culture-independent methods may help identify imbalances in milk microbial ecology associated with this often debilitating condition.

The potential effects of the richness of milk microbiota reported in this study on infant gut colonization are intriguing. Clearly, human milk consumption introduces the infant to hundreds of phylotypes of bacteria that have a direct route to the gastrointestinal tract. Exposure of the breastfed infant to the bacterial richness in milk may be one factor contributing to the differential fecal microbiota between breastfed and formula-fed infants [28]. Additionally, ingestion of such a wealth of bacterial phylotypes may also contribute to the protective effects of breastfeeding against diarrheal [1] and respiratory [2] disease, and reduced risk of developing obesity [3], [4].

In summary, results from this study have confirmed that human milk bacterial communities are highly diverse and complex. A logical next step will be to investigate a larger population to determine which characteristics, if any, of the milk microbiome are associated with enhanced health outcomes for women and their infants. Likewise, it is of interest to determine if factors such as maternal race, parity, mode of delivery, and maternal diet influence these community characteristics. Previous studies have shown that the microbiota present in the lower gastrointestinal tract [29], vagina [30] and oral cavity [31], and more importantly the differential composition of these communities in healthy versus diseased states, are related to the health of the human host. The application of these principles to human milk may have important implications for mammary health, bacterial colonization of the infant's gastrointestinal tract, and short- and long-term infant health.

## Materials and Methods

### Participants and sample collection

Breastfeeding women (*n* = 16) were recruited from Pullman, WA and the surrounding area. Inclusion criteria required being self-reported as healthy, between 20–40 yr of age, and nursing ≥5 times/d. Procedures were carried out under the approval of the Washington State University Institutional Review Board and the University of Idaho Human Assurances Committee.

Participants elected one breast from which to donate samples for the duration of the study and were asked not to nurse or express milk from that breast during the 2 h prior to sample donation. Three samples were collected from each woman between 0700 and 1000 h with a 1–2 wk interval between samples. On each sample collection day, participants completed a short health questionnaire describing unusual health- or lactation- related problems. With the exception of one woman who was not able to donate one sample due to non-health related circumstances, each participant donated 3 samples.

Before sample collection, the breast was cleaned with an iodine swab to reduce bacteria residing on the skin, and breast milk was collected with a Hygienikit® sterile milk collection unit (Ameda, Cary, IN) and an electric breast pump. To ensure the collection of a “complete” expression, participants continued to pump until flow had subsided. The somatic cell count of all samples was analyzed using the Delaval somatic cell counter (Delaval, Tumba, Sweden). Samples were immediately frozen and stored at −20°C until further analysis.

### DNA isolation and PCR amplification

DNA extraction was performed as previously described [32]. Briefly, 0.5 mL aliquots of each milk sample were submitted to an enzymatic lysis with lysozyme (500 µg), mutanolysin (50 µg), and lysostaphin (4 µg) and incubated at 37°C for 1 h. Mechanical lysis was then performed by bead-beating with 0.1 mm diameter zirconia-silica beads (Biospec) using the FastPrep machine (MP Biomedicals). DNA was then extracted with the QIAamp DNA Mini Kit (Qiagen) according to the manufacturer's instructions. The QIAamp protocol was modified slightly with the addition of 100 µL of 3 M sodium acetate to the milk lysate before passing it through the QIAamp mini spin column. This additional step lowered the pH of the milk lysate below 7.0 to allow for maximum DNA binding to the silica column lending to an overall improved DNA yield.

PCR reactions were performed using the universal primer set 27F and 338R to amplify the V1–V2 hypervariable segment of the 16S rRNA gene resulting in ∼300 base pair amplicons which are suitable for phylogenetic classification of bacteria [33]. The primer sequences were as follows:

27F- GCCTTGCCAGCCCGCTCAGTC **AGAGTTTGATCCTGGCTCAG**, and

338R- GCCTCCCTCGCGCCATCAGTGNNNNNNNNCA**TGCTGCCTCCCGTAGGAGT**.

The 454 Life Sciences® primers B and A are underlined in the 27F and 338R primers respectively, whereas the universal primers 27F and 338R are bolded. The series of Ns in the 338R primer denotes the location of the eight base pair barcode unique to each sample embedded in the primer set for each sample as previously described [13].

PCR reactions were prepared in a hood that was UV sterilized for 30 min before the introduction of reagents or samples, and a negative control with no template added was carried out alongside each sample for every primer set that was utilized. Each 50-µL PCR reaction was carried out with reagents supplied by Applied Biosystems including 0.5 µL of both forward and reverse primers (10 µM), 5 µL 10× PCR buffer, 6 µL MgCl (25 mM), 2.5 µL DMSO, 0.4 µL dNTP mix (25 mM), and 0.2 µL AmpliTaq® DNA polymerase. Reactions were initially carried out adding 2 µL DNA template, and later repeated adding 5 µL template for those samples which did not generate a PCR product with 2 µL template added. Thermal cycler settings included a 5 min denaturation step at 94°C followed by 35 cycles of 94°C for 1 min, 55°C for 1 min, and 72°C for 2 min. A final elongation step at 72°C for 2 min was then performed to complete each reaction before storing PCR products at −20°C until further use.

The efficiency of the reactions and the absence of contamination in the negative controls were then verified by electrophoresis of the PCR product on a 1% agarose gel, staining with ethidium bromide and UV exposure. A 10-µL aliquot of each PCR reaction was then added to a pool sample that underwent emulsion PCR as previously described [34]. Pyrosequencing was performed at the Institute for Genome Sciences at the University of Maryland School of Medicine on a 454 Life Sciences Genome Sequencer FLX machine (Roche).

### Bioinformatics and statistical analysis

Mothur [35] was utilized to bin sequences by sample and carry out quality control procedures. Although the occurrence of sequencing error in data sets generated with 454 pyrosequencing has been reported to be only 0.5% [36], these errors significantly inflate the number of observed OTUs [37]. To minimize these effects, a conservative approach was adopted and the data were subjected to a rigorous quality control procedure. Sequences were removed from the data set if they had any ambiguous bases, contained homopolymer runs greater than 8 bases, greater than one difference from the barcode, or greater than 2 differences from the forward primer. Sequences were also removed if they did not maintain an average quality score of 35 over a sliding window of 50 bases. Sequences were then subjected to a pairwise alignment using Smith-Waterman global alignment against Mothur's Silva reference database, and were trimmed to cover the same region. Sequences that did not align correctly were then removed from the dataset. A 2% single-linkage, precluster method was then employed as it has been shown to remove sequences that may contain sequencing error [38]. Potential chimeras were identified and removed using Mothur's implementation of the ChimeraSlayer algorithm from the Broad Institute.

Over 160,000 sequence reads passed quality control measures, although roughly 40% of the original sequences did not. From 1,100 to 8,500 sequences were acquired for each sample with an average of 3,400 per sample. To determine phylogeny sequences were organized into likely phylotype assignments at the genus level using The Ribosomal Data Base Project Bayesian classifier [39]. For OTU-based analyses the average neighbor clustering algorithm was utilized to group sequences at a 3% similarity level. The R package OTUBase [40] was utilized to cluster samples based on the Bray-Curtis similarity index using complete linkage clustering and generate the dendrogram. The collective “core OTUs” were assigned to taxonomic groups by comparing one representative sequence from each OTU against the Mothur SILVA SEED database using the bayesian method for assignment to the most likely phylogeny.

### Culture dependent bacterial analysis

Culture-based bacterial analysis was performed following the guidelines outlined by the National Mastitis Council Laboratory and Field Handbook on Bovine Mastitis [26]. Briefly, 50 µL of each sample was spread onto a blood agar plate and incubated aerobically at 37°C for 48 h. Bacteria were identified as coagulase-positive or –negative *Staphylococci*, *Streptococcus*, *Corynebacterium*, and coliforms based on colony morphology, hemolytic activity, reaction on Christie, Atkins and Munch-Peterson-esculin (CAMP) plates, catalase production, coagulase test reactions and Gram staining.

## Supporting Information

Table S1 **The total number of sequence reads for each of the 15 most abundant genera in the milk.** **A.** Sequence reads are listed for subjects 1–4. **B.** Sequence reads are listed for subjects 5–8. **C.** Sequence reads are listed for subjects 9–12. **D.** Sequence reads are listed for subjects 13–16.
(DOCX)
