# Supplementary material for: Characterization of the Diversity and Temporal Stability of Bacterial Communities in Human Milk
Source: PLoS One. 2011 Jun 17;6(6):e21313. doi: 10.1371/journal.pone.0021313 (PMC3117882; doi:10.1371/journal.pone.0021313)
Supplement: Table S1 — The total number of sequence reads for each of the 15 most abundant genera in the milk. A. Sequence reads are listed for subjects 1–4. B. Sequence reads are listed for subjects 5–8. C. Sequence reads are listed for subjects 9–12. D. Sequence reads are listed for subjects 13–16. (DOCX) [file pone.0021313.s001.docx]

| **Table S1A.** The total number of sequence reads for each of the 15 most abundant genera in milk from subjects 1 to 4 | | | | | | | | | | | | |
| --- | --- | --- | --- | --- | --- | --- | --- | --- | --- | --- | --- | --- |
|  | Subject Identification and Time-point | | | | | | | | | | | |
|  | S1 | | | S2 | | | S3 | | | S4 | | |
|  | 1 | 3 | 4 | 1 | 3 | 4 | 1 | 3 | 4 | 1 | 3 | 4 |
| *Streptococcus* | 215 | 225 | 118 | 224 | 1188 | 832 | 1023 | 1077 | 928 | 540 | 769 | 341 |
| *Staphylococcus* | 59 | 48 | 23 | 281 | 556 | 162 | 306 | 231 | 444 | 1436 | 748 | 1398 |
| *Serratia* | 551 | 249 | 637 | 108 | 53 | 13 | 323 | 189 | 437 | 104 | 283 | 188 |
| *Corynebacterium* | 76 | 28 | 12 | 297 | 912 | 519 | 29 | 41 | 79 | 236 | 57 | 81 |
| *Pseudomonas* | 332 | 205 | 293 | 59 | 27 | 5 | 177 | 136 | 208 | 90 | 230 | 105 |
| *Ralstonia* | 682 | 266 | 244 | 33 | 24 | 8 | 107 | 58 | 19 | 388 | 115 | 64 |
| *Propionibacterium* | 82 | 44 | 30 | 45 | 10 | 4 | 242 | 159 | 165 | 68 | 228 | 35 |
| *Sphingomonas* | 309 | 82 | 125 | 31 | 4 | 8 | 35 | 36 | 70 | 33 | 72 | 17 |
| *Rothia* | 0 | 19 | 5 | 13 | 137 | 93 | 143 | 133 | 128 | 17 | 16 | 14 |
| *Bradyrhizobium* | 173 | 165 | 120 | 30 | 13 | 3 | 38 | 25 | 57 | 36 | 48 | 43 |
| *Gemella* | 4 | 34 | 0 | 14 | 104 | 18 | 94 | 13 | 21 | 665 | 64 | 130 |
| *Veillonella* | 30 | 16 | 0 | 17 | 123 | 49 | 7 | 19 | 3 | 3 | 50 | 70 |
| *Actinomyces* | 18 | 1 | 30 | 4 | 65 | 52 | 54 | 55 | 22 | 3 | 3 | 0 |
| *Granulicatella* | 23 | 0 | 0 | 13 | 175 | 44 | 3 | 38 | 4 | 3 | 3 | 2 |
| *Prevotella* | 20 | 0 | 1 | 13 | 230 | 54 | 8 | 10 | 11 | 0 | 13 | 1 |
| Other | 626 | 322 | 297 | 283 | 374 | 139 | 544 | 420 | 331 | 277 | 349 | 169 |
| Total | 3200 | 1704 | 1935 | 1465 | 3995 | 2003 | 3133 | 2640 | 2927 | 3899 | 3048 | 2658 |

| **Table S1B.** The total number of sequence reads for each of the 15 most abundant genera in milk from subjects 6 to 8 | | | | | | | | | | | | |
| --- | --- | --- | --- | --- | --- | --- | --- | --- | --- | --- | --- | --- |
|  | Subject Identification and Time-point | | | | | | | | | | | |
|  | S5 | | | S6 | | | S7 | | | S8 | | |
|  | 1 | 3 | 4 | 1 | 3 | 4 | 1 | 3 | 4 | 1 | 3 | 4 |
| *Streptococcus* | 360 | 1948 | 370 | 55 | 601 | 410 | 1568 | 1253 | 1092 | 95 | 48 | 480 |
| *Staphylococcus* | 5140 | 1935 | 7520 | 71 | 327 | 247 | 114 | 336 | 99 | 669 | 859 | 1147 |
| *Serratia* | 20 | 97 | 21 | 540 | 1255 | 1274 | 176 | 215 | 221 | 72 | 44 | 421 |
| *Corynebacterium* | 591 | 686 | 527 | 19 | 22 | 28 | 26 | 48 | 9 | 410 | 273 | 768 |
| *Pseudomonas* | 1 | 75 | 9 | 390 | 632 | 756 | 143 | 122 | 158 | 48 | 19 | 212 |
| *Ralstonia* | 3 | 93 | 6 | 36 | 62 | 54 | 70 | 353 | 524 | 26 | 19 | 127 |
| *Propionibacterium* | 4 | 6 | 2 | 53 | 184 | 61 | 83 | 87 | 134 | 180 | 104 | 1403 |
| *Sphingomonas* | 2 | 21 | 9 | 335 | 577 | 541 | 55 | 29 | 45 | 2 | 8 | 50 |
| *Rothia* | 14 | 78 | 18 | 45 | 98 | 268 | 14 | 86 | 200 | 11 | 11 | 163 |
| *Bradyrhizobium* | 3 | 21 | 2 | 211 | 466 | 430 | 10 | 15 | 22 | 3 | 4 | 19 |
| *Gemella* | 5 | 9 | 5 | 2 | 21 | 4 | 0 | 38 | 64 | 0 | 0 | 1 |
| *Veillonella* | 3 | 42 | 14 | 0 | 8 | 7 | 9 | 11 | 109 | 3 | 0 | 15 |
| *Actinomyces* | 1 | 8 | 1 | 0 | 1 | 0 | 3 | 1 | 17 | 0 | 0 | 2 |
| *Granulicatella* | 0 | 0 | 0 | 0 | 4 | 1 | 1 | 4 | 121 | 7 | 0 | 3 |
| *Prevotella* | 0 | 0 | 0 | 1 | 8 | 6 | 10 | 20 | 85 | 0 | 1 | 0 |
| Other | 20 | 60 | 42 | 437 | 633 | 781 | 187 | 384 | 376 | 91 | 61 | 265 |
| Total | 6167 | 5079 | 8546 | 2195 | 4899 | 4868 | 2469 | 3002 | 3276 | 1617 | 1451 | 5076 |

| **Table S1C.** The total number of sequence reads for each of the 15 most abundant genera in milk from subjects 9 to 12 | | | | | | | | | | | |
| --- | --- | --- | --- | --- | --- | --- | --- | --- | --- | --- | --- |
|  | Subject Identification and Time-point | | | | | | | | | | |
|  | S9 | | | S10 | | | S11 | | S12 | | |
|  | 1 | 3 | 4 | 1 | 3 | 4 | 1 | 3 | 1 | 3 | 4 |
| *Streptococcus* | 626 | 434 | 1658 | 1066 | 663 | 1465 | 1047 | 2459 | 95 | 105 | 147 |
| *Staphylococcus* | 965 | 1792 | 1597 | 786 | 389 | 579 | 369 | 450 | 108 | 109 | 237 |
| *Serratia* | 368 | 151 | 96 | 297 | 219 | 166 | 284 | 143 | 348 | 296 | 578 |
| *Corynebacterium* | 74 | 521 | 647 | 16 | 24 | 40 | 28 | 23 | 55 | 25 | 57 |
| *Pseudomonas* | 258 | 66 | 43 | 167 | 191 | 93 | 320 | 139 | 204 | 170 | 232 |
| *Ralstonia* | 214 | 41 | 34 | 117 | 425 | 214 | 36 | 22 | 18 | 105 | 284 |
| *Propionibacterium* | 192 | 80 | 66 | 167 | 445 | 172 | 186 | 98 | 168 | 147 | 280 |
| *Sphingomonas* | 69 | 28 | 13 | 62 | 78 | 53 | 282 | 134 | 36 | 5 | 38 |
| *Rothia* | 104 | 95 | 454 | 90 | 99 | 95 | 115 | 217 | 5 | 22 | 20 |
| *Bradyrhizobium* | 10 | 7 | 5 | 35 | 90 | 13 | 221 | 86 | 15 | 28 | 21 |
| *Gemella* | 14 | 17 | 34 | 44 | 34 | 26 | 139 | 47 | 9 | 0 | 3 |
| *Veillonella* | 43 | 151 | 114 | 61 | 59 | 73 | 80 | 42 | 4 | 4 | 25 |
| *Actinomyces* | 9 | 5 | 4 | 12 | 19 | 10 | 88 | 43 | 5 | 9 | 13 |
| *Granulicatella* | 1 | 0 | 12 | 38 | 20 | 22 | 48 | 6 | 0 | 5 | 15 |
| *Prevotella* | 7 | 38 | 8 | 0 | 1 | 1 | 40 | 5 | 19 | 0 | 4 |
| Other | 448 | 227 | 118 | 338 | 521 | 261 | 1082 | 552 | 595 | 1577 | 396 |
| Total | 3402 | 3653 | 4903 | 3296 | 3277 | 3283 | 4365 | 4466 | 1684 | 2607 | 2350 |

| **Table S1D.** The total number of sequence reads for each of the 15 most abundant genera in milk from subjects 13 to 16 | | | | | | | | | | | | |
| --- | --- | --- | --- | --- | --- | --- | --- | --- | --- | --- | --- | --- |
|  | Subject Identification and Time-point | | | | | | | | | | | |
|  | S13 | | | S14 | | | S15 | | | S16 | | |
|  | 1 | 3 | 4 | 1 | 3 | 4 | 1 | 3 | 4 | 1 | 3 | 4 |
| *Streptococcus* | 4478 | 363 | 399 | 1820 | 979 | 1408 | 1864 | 1126 | 2226 | 120 | 45 | 73 |
| *Staphylococcus* | 76 | 1638 | 273 | 24 | 85 | 95 | 534 | 1727 | 1138 | 495 | 3687 | 435 |
| *Serratia* | 23 | 129 | 25 | 152 | 245 | 650 | 61 | 191 | 45 | 238 | 176 | 433 |
| *Corynebacterium* | 7 | 47 | 49 | 14 | 9 | 17 | 107 | 67 | 75 | 1714 | 330 | 312 |
| *Pseudomonas* | 22 | 209 | 24 | 102 | 102 | 347 | 41 | 130 | 37 | 132 | 133 | 302 |
| *Ralstonia* | 9 | 14 | 26 | 202 | 14 | 477 | 18 | 54 | 16 | 100 | 16 | 333 |
| *Propionibacterium* | 5 | 102 | 22 | 23 | 50 | 173 | 53 | 69 | 31 | 48 | 10 | 71 |
| *Sphingomonas* | 14 | 138 | 34 | 65 | 73 | 215 | 6 | 65 | 18 | 95 | 58 | 91 |
| *Rothia* | 0 | 22 | 19 | 277 | 59 | 189 | 473 | 50 | 298 | 0 | 1 | 11 |
| *Bradyrhizobium* | 14 | 130 | 35 | 44 | 71 | 225 | 6 | 29 | 2 | 64 | 63 | 98 |
| *Gemella* | 0 | 1 | 2 | 268 | 78 | 185 | 113 | 101 | 441 | 0 | 0 | 0 |
| *Veillonella* | 2 | 42 | 27 | 146 | 60 | 54 | 379 | 183 | 431 | 8 | 0 | 0 |
| *Actinomyces* | 0 | 0 | 4 | 81 | 8 | 32 | 765 | 47 | 118 | 1 | 1 | 0 |
| *Granulicatella* | 0 | 0 | 4 | 92 | 114 | 159 | 83 | 29 | 208 | 0 | 0 | 0 |
| *Prevotella* | 1 | 38 | 31 | 21 | 63 | 91 | 209 | 32 | 118 | 4 | 1 | 1 |
| Other | 29 | 438 | 131 | 203 | 244 | 667 | 796 | 227 | 615 | 202 | 111 | 2635 |
| Total | 4680 | 3311 | 1105 | 3534 | 2254 | 4984 | 5508 | 4127 | 5817 | 3221 | 4632 | 4795 |
